# Wernicke's Encephalopathy in a Patient With Type III Intestinal Failure: A Case Report

**DOI:** 10.7759/cureus.44975

**Published:** 2023-09-10

**Authors:** Daniel González-Arroyave, Jaime A Ramírez-Arbeláez, Luis M Barrera-Lozano, Cristian L Muñoz, Juanita Restrepo-Arevalo, Ana Jaillier, Veronica Hurtado, Carlos M Ardila

**Affiliations:** 1 Surgery, Universidad Pontificia Bolivariana, Medellín, COL; 2 Transplant, Hospital San Vicente Fundación, Rionegro, COL; 3 Transplant, Universidad de Antioquia, Medellín, COL; 4 Medicine, Universidad Pontificia Bolivariana, Medellín, COL; 5 Nutrition, Hospital San Vicente Fundación, Rionegro, COL; 6 Rehabilitation Medicine, Hospital San Vicente Fundación, Rionegro, COL; 7 Basic Sciences, Universidad de Antioquia, Medellín, COL

**Keywords:** case study, emergency services, thiamine deficiency, intestinal failure, wernicke encephalopathy

## Abstract

This case is about a 38-year-old male patient with a history of type III intestinal failure due to chronic intestinal pseudo-obstruction caused by gastrointestinal dysmotility, cardiac and intestinal arrhythmia syndrome, dependence on parenteral nutrition, sinus dysfunction, and carrying a pacemaker. The patient presented with symptoms suggestive of a new episode of intestinal obstruction. A contrast-enhanced abdominal computed tomography scan was performed, revealing intestinal obstruction with a transition zone in the jejunum. Non-operative medical management of the obstructive condition was initiated. However, after 10 days of medical management, the patient began experiencing nausea and dizziness. Initially, symptomatic management was provided, but the patient reported persistent vertigo-like sensations. Following evaluation by multiple specialties, magnetic resonance imaging (MRI) was requested, which showed bilateral and symmetrical hyperintensity on T2-weighted images of the dorsomedial aspect of the thalami around the third ventricle, in the periaqueductal gray matter, the mesencephalic tectum, and, to a lesser extent, the bulbar tectum, findings suggestive of Wernicke's encephalopathy. Urgent intravenous thiamine replacement was initiated. After 10 days of effective treatment, the patient exhibited a nearly complete improvement in symptoms. A follow-up MRI was ordered, indicating considerable improvement when compared to the previous study.

## Introduction

Type III intestinal failure is a severe ailment characterized by the gut's incapacity to absorb macronutrients, water, and electrolytes [1]. Its occurrence varies between 5 to 20 instances per one million people, with even higher rates observed in developing and non-developing economies [2]. The causes encompass a range of conditions, with short bowel syndrome (SBS) and chronic intestinal pseudo-obstruction (CIPO) being the prevailing factors. These two factors are the most prevalent in both adult (74.7% for SBS and 18% for CIPO) and pediatric (52.4% for SBS and 22.9% for CIPO) cases [1,3].

Intestinal pseudo-obstruction is a condition that accounts for approximately 9.7% of type III intestinal failure in the adult population. Most cases are due to idiopathic causes or systemic diseases (infections, autoimmune disorders, Duchenne muscular dystrophy, and degenerative neuropathies, among others), which constitute 50% of adult cases [4]. The clinical course is characterized by exacerbations, which can be triggered by various factors such as viral or bacterial infections, central line sepsis, psychological stress, and malnutrition. In more severe cases, the natural progression of the disease can ultimately lead to intestinal insufficiency [4,5].

Wernicke's encephalopathy is a neuropsychiatric syndrome characterized by nystagmus and ophthalmoplegia, changes in mental status, and instability in posture and gait. This triad is observed in only 10% of patients [6]. This condition has a mortality rate of 17% and is more common in men than in women [4]. In adults, it is estimated that only about 15% of cases are diagnosed before death [5]. The prevalence of Wernicke's encephalopathy in cases of intestinal obstruction, including type III obstruction, would depend on various factors such as the patient's overall health, nutrition status, and underlying conditions [4-6]. Most Wernicke's encephalopathy cases occur in individuals with thiamine deficiency, although in the United States, the most associated factor is alcoholism. Other factors include nutritional deficiency, hyperemesis gravidarum, intestinal obstruction, and malignancy [5].

This case is presented with the aim of describing the diagnosis and treatment of a patient with a history of type III intestinal failure who developed Wernicke's encephalopathy.

## Case presentation

The case described a 38-year-old male patient with a history of type III intestinal failure due to chronic intestinal pseudo-obstruction caused by gastrointestinal dysmotility, cardiac and intestinal arrhythmia syndrome, dependence on parenteral nutrition, sinus dysfunction, and a pacemaker. The patient had a history of chronic intestinal insufficiency due to a motor disorder of the intestines. For several years, the patient has been enrolled in an intestinal rehabilitation program that necessitates regular monitoring of nutritional, biometric, and electrolytic parameters. In fact, the patient was admitted to a high-complexity hospital with a clinical presentation of diffuse, mild-intensity abdominal pain lasting for six days, along with multiple vomiting episodes. There was no fever, diarrhea, jaundice, or other associated symptoms.

Upon admission, the patient's vital signs were as follows: blood pressure 98/54 mmHg, heart rate 67 per minute, respiratory rate 16 per minute, and oxygen saturation 95%. The physical examination revealed an alert patient who was oriented in all three mental spheres, with mild dehydration, minimal tenderness upon abdominal palpation, and no clear signs of peritoneal irritation.

**Figure 1:**
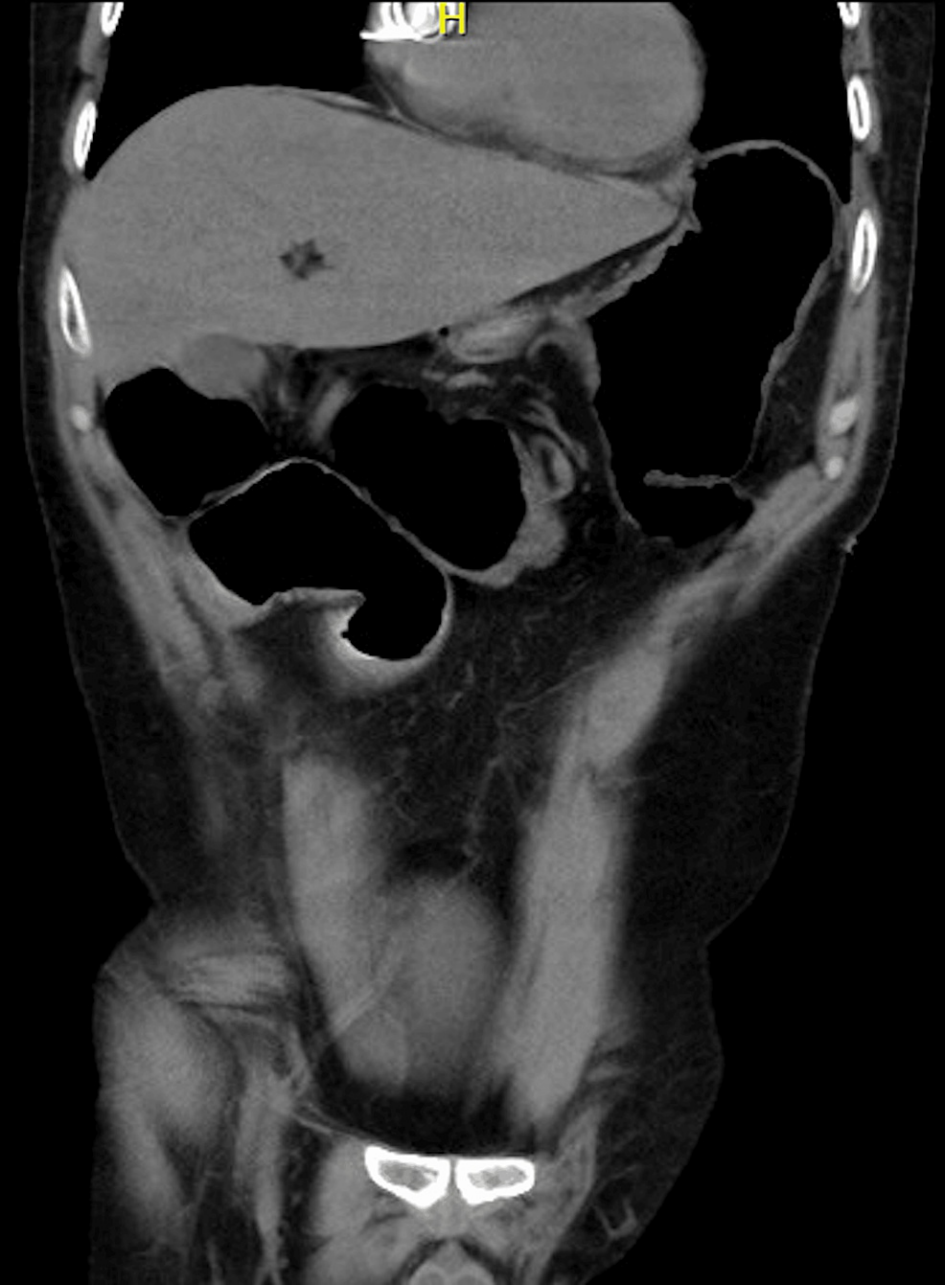
This coronal section of the abdominal CT scan shows extensive dilatation of slender loops from the duodenum.

The patient presented with a clinical picture suggestive of a new episode of intestinal obstruction. A contrast-enhanced abdominal CT scan showed intestinal obstruction with a transition zone in the jejunum (Figure 1).

There were no signs of complications or intestinal loop suffering. Non-operative medical management of the obstructive condition was initiated.

After 10 days of medical management of intestinal obstruction, with appropriate improvement and no elevation of acute phase reactants, the patient began experiencing nausea and dizziness. Initially, symptomatic management was provided; however, the patient reported persistent vertigo-like sensations, prompting consultation with otolaryngology to rule out peripheral origin vertigo. Otolaryngology suggested continuing the search for systemic pathology as peripheral origin vertigo was ruled out. Consequently, an electrophysiology assessment was requested, considering the patient's pacemaker history, as dysfunction of the pacemaker could be a potential cause of the dizziness. The pacemaker was then reprogrammed. However, the patient continued to experience persistent dizziness without improvement and reported a new symptom: progressive vision impairment. As a result, a neurology consultation was requested.

Upon evaluation by neurology, the patient exhibited blurred vision, described the dizziness as a spinning sensation with photophobia, and showed nystagmus on neurological examination, specifically horizontal binocular nystagmus with a fast phase to the left that worsened with gaze fixation and changed direction with extreme gaze. Additionally, there was right upper limb dysmetria, suggestive of a cerebellar syndrome. Therefore, MRI was requested.

**Figure 2:**
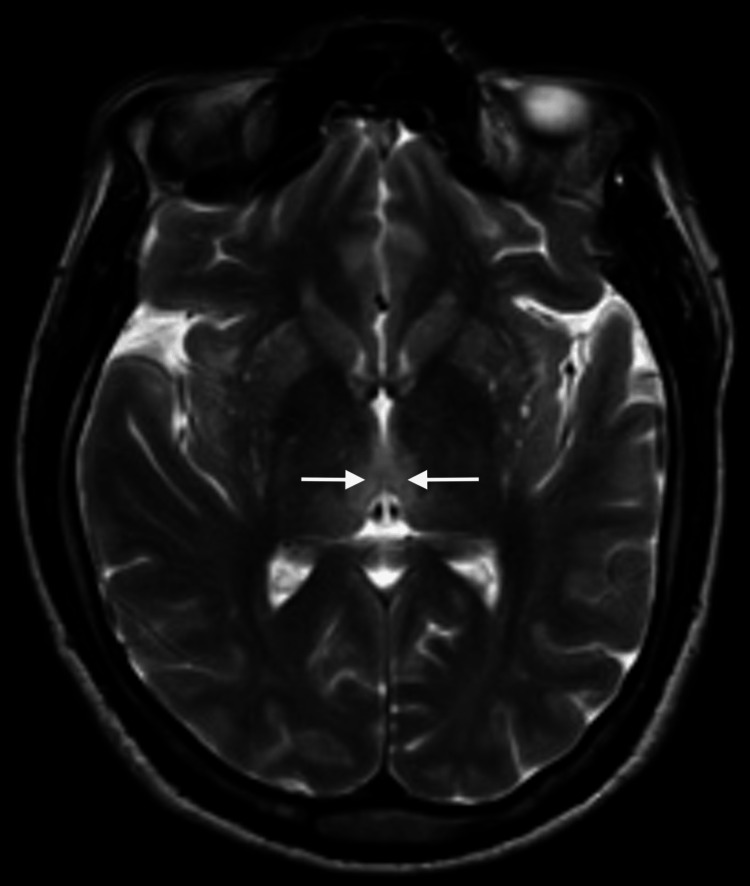
Contrast-enhanced MRI: An initial study. Axial T2 thalami. Bilateral and symmetrical hyperintensity of the dorsomedial aspect of the thalami is observed on T2-weighted images (arrows).

**Figure 3:**
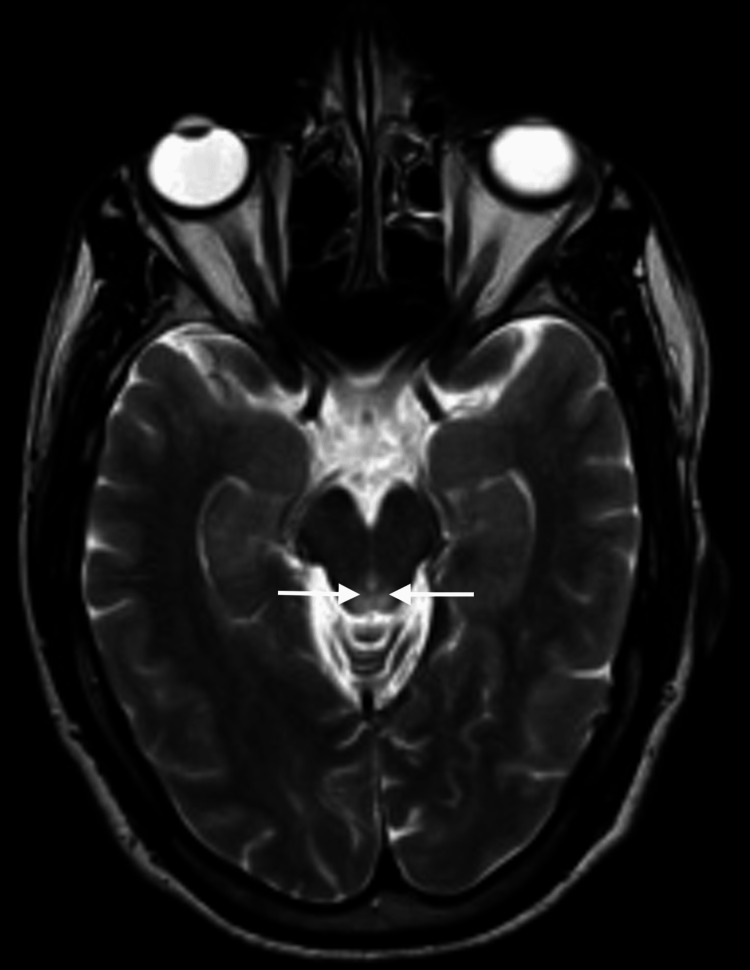
Contrast-enhanced MRI: An initial study. Axial T2. Periaqueductal bilateral and symmetrical hyperintensity around the third ventricle, in the periaqueductal gray matter, is observed on T2-weighted images (arrows).

**Figure 4:**
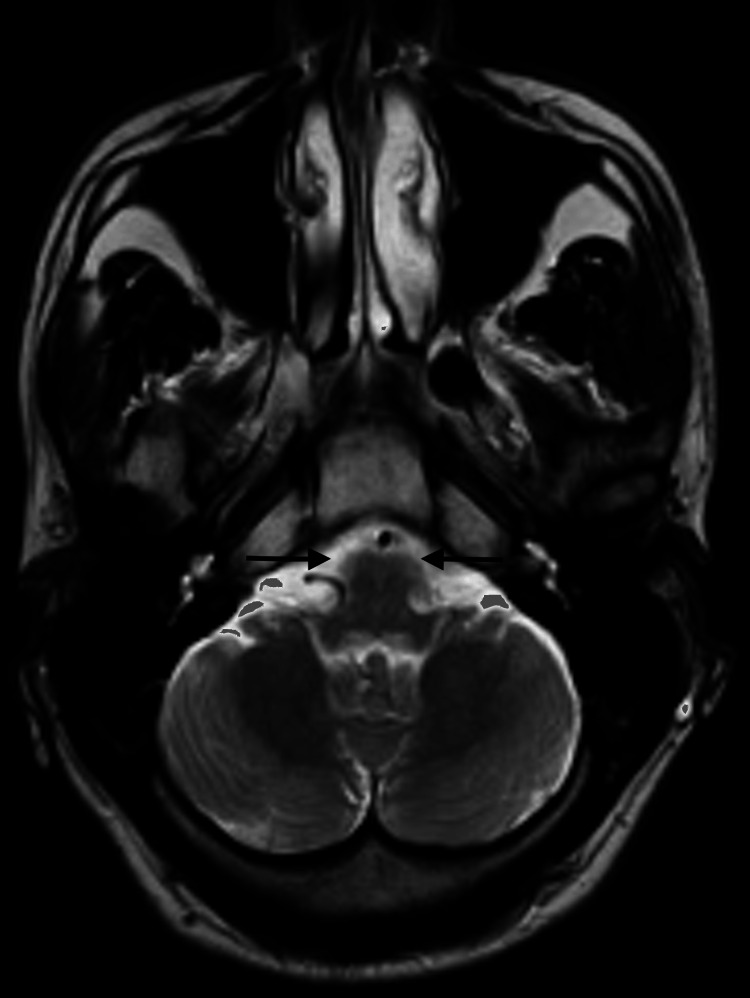
Contrast-enhanced MRI: An initial study. Axial T2 bulb. Bilateral and symmetrical hyperintensity of the mesencephalic tectum and, to a lesser extent, the bulbar tectum is observed on T2-weighted images (arrows).

Three days passed from the initial neurology evaluation, and the patient reported a significant progression of symptoms, including pronounced drowsiness with rapidly progressive dementia, vertigo, ataxia, and ophthalmoparesis. The MRI results indicated bilateral and symmetrical hyperintensity on T2-weighted images of the dorsomedial aspect of the thalami around the third ventricle, in the periaqueductal gray matter, the mesencephalic tectum, and, to a lesser extent, the bulbar tectum. These findings were suggestive of Wernicke's encephalopathy (Figures 2-4).

Urgent intravenous thiamine replacement was initiated (IV thiamine 500 mg infusion over 30 minutes for three consecutive days, followed by 250 mg IV every 24 hours).

**Figure 5:**
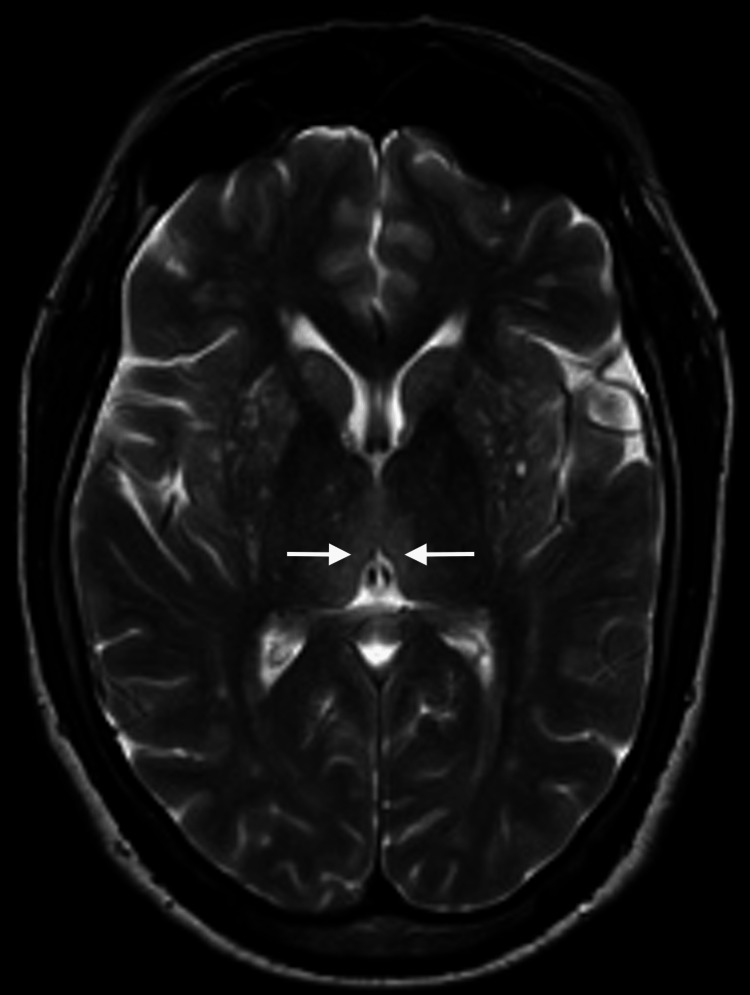
Contrast-enhanced MRI: Follow-up study. Axial T2 thalami follow-up. Slight improvement in bilateral and symmetrical hyperintensity of the dorsomedial aspect of the thalami is observed on T2-weighted images (arrows).

**Figure 6:**
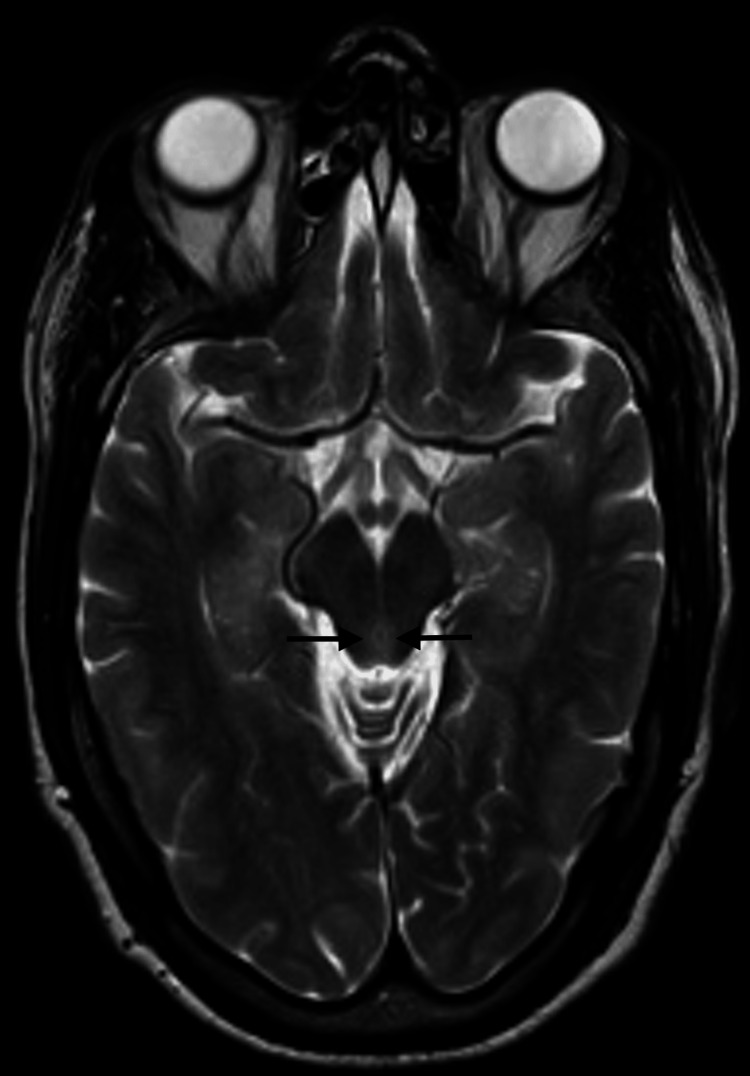
Contrast-enhanced MRI: Follow-up study. Axial T2 periaqueductal follow-up. Slight improvement in bilateral and symmetrical hyperintensity around the third ventricle in the periaqueductal gray matter is observed on T2-weighted images (arrows).

**Figure 7:**
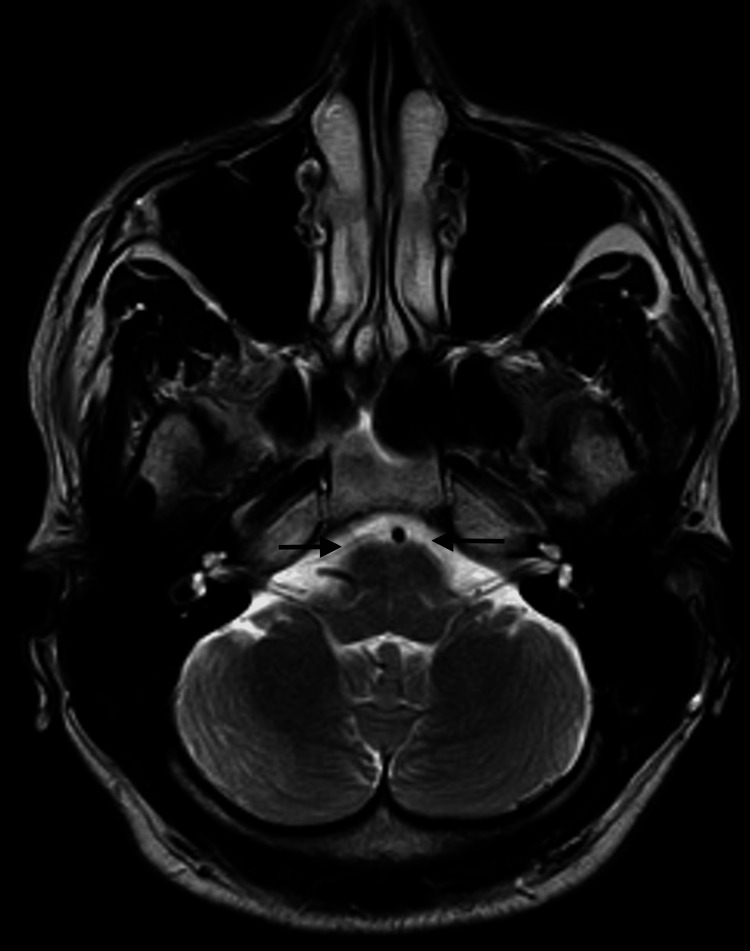
Contrast-enhanced MRI: A follow-up study. Axial T2 bulb follow-up. Slight improvement in bilateral and symmetrical hyperintensity of the mesencephalic tectum and, to a lesser extent, the bulbar tectum is observed on T2-weighted images (arrows).

After four days of treatment, the patient began to experience improvements in cognitive function and ocular paralysis. After 10 days of effective treatment, the patient exhibited nearly complete symptom improvement. A follow-up MRI was ordered, revealing considerable improvement compared to the previous study (Figures 5-7).

As soon as the patient improved from his obstructive condition, the oral route was started again; however, considering that, due to his intestinal motor disorder, the oral route is not sufficient to cover the patient's protein-calorie requirements, the indefinite administration of parenteral nutrition is required in addition to a prophylactic administration of thiamine due to the high risk of this patient’s return to deficiency encephalopathy. The patient continued with outpatient management and follow-ups with intestinal rehabilitation surgery and neurology. 

## Discussion

Chronic intestinal insufficiency is a condition that arises when intestinal function is diminished below the level necessary for proper absorption of nutrients, water, and electrolytes, necessitating intravenous supplementation to maintain health and growth. Five pathophysiological conditions causing chronic intestinal insufficiency have been identified, including short bowel, intestinal fistula, impaired intestinal motility, mechanical obstruction, and extensive mucosal disease [7].

In the present case, the patient had a history of chronic intestinal insufficiency due to a motor disorder of the intestines. For several years, the patient has been enrolled in an intestinal rehabilitation program that necessitates regular monitoring of nutritional, biometric, and electrolytic parameters. However, the patient was admitted to the emergency department because individuals with this type of condition have multiple risk factors that can lead to frequent relapses and hospital readmissions due to obstructive episodes.

Wernicke's encephalopathy is a neurological disorder characterized by alterations in mental status, nystagmus, ophthalmoplegia, and changes in posture. However, its diagnosis is challenging if not in the hands of specialized medical personnel, and it is estimated that only around 15% of cases are diagnosed before death. The disease is caused by thiamine deficiency, which, in the patient's case, occurs due to the intestine's inability to absorb it properly [8]. Thiamine deficiency triggers Wernicke's encephalopathy through its essential role in various biochemical pathways that are critical for normal brain function. Thiamine, also known as vitamin B1, is a water-soluble vitamin that plays a key role in energy metabolism and maintaining the integrity of the nervous system. Wernicke's encephalopathy develops when the brain does not receive adequate thiamine, leading to a disruption in these vital processes [6,8]. Thiamine is a coenzyme for several enzymes involved in carbohydrate metabolism, particularly in the citric acid cycle (also known as the Krebs cycle) and the pentose phosphate pathway. These pathways are essential for the generation of adenosine triphosphate (ATP), the primary energy currency of cells. Thiamine deficiency impairs the proper functioning of these enzymes, leading to reduced ATP production. The brain is highly energy-dependent, and inadequate energy production can result in neuronal dysfunction and damage [6]. The precise reason for brain injury in Wernicke's encephalopathy remains uncertain; however, it could be linked to localized lactic acid buildup, breakdown of the protective blood-brain barrier, excessive excitation of neural cells, inflammation, or inadequate cellular ATP levels [8].

The patient's chronic intestinal insufficiency, which led to a chronic thiamine deficiency, resulted in the development of Wernicke's encephalopathy with all the aforementioned symptoms. It is important to highlight that although the patient has a chronic thiamine deficiency due to malabsorption in the intestine, in the last two years, there has been a phenomenon of drug shortages in our country, probably because of the SARS-CoV-2 pandemic. Among these drugs are multivitamin vials, which are routinely placed in patients dependent on parenteral nutrition. This same situation has been reported in different health systems around the world [9]. Therefore, we consider that these two circumstances caused the patient to present this deficiency. Following the onset of symptoms and evaluation by specialized medical personnel, contrast-enhanced MRI was requested, revealing bilateral and symmetrical hyperintensity in regions such as the thalamus, third ventricle, periaqueductal region, and bulb, confirming the diagnosis of thiamine-deficient Wernicke's encephalopathy.

Once the diagnosis of thiamine-deficient Wernicke's encephalopathy was confirmed, immediate intravenous thiamine replacement was initiated, leading to a progressive improvement in the patient's symptoms. A subsequent follow-up MRI showed significant improvement in the previously identified findings, indicating the effectiveness of the treatment.

Wernicke's encephalopathy is still a clinical diagnosis and a medical emergency that necessitates rapid treatment if it is suspected. Laboratory testing or imaging investigations should not be used to delay treatment [10]. Neuroimaging is not the ultimate diagnostic approach since it lacks enough sensitivity, as demonstrated by Isenberg-Grzeda et al.’s research on non-alcoholic Wernicke's encephalopathy, in which MRI provided positive results in just three out of 10 cases [11]. The European Federation of Neurological Societies and the Royal College of Physicians recommend giving 500 mg of parenteral thiamine three times per day until the symptoms of acute Wernicke's encephalopathy disappear [12]. Lower doses have failed to generate sufficient improvement in certain people in some circumstances, and oral absorption is unreliable; therefore, it is not recommended. Long-term prophylaxis with 50-100 mg of thiamine three times per day is advised until the patient is no longer deemed at risk [13].

## Conclusions

Wernicke's encephalopathy is a serious medical condition that can affect patients with a history of prolonged parenteral nutrition and other risk factors. It is crucial for physicians to be aware of the symptoms of this disease and to consider thiamine administration in patients suspected of having Wernicke's encephalopathy to prevent severe complications.
